# A putative RNA binding protein from *Plasmodium vivax* apicoplast

**DOI:** 10.1002/2211-5463.12351

**Published:** 2017-12-31

**Authors:** Sofía M. García‐Mauriño, Antonio Díaz‐Quintana, Francisco Rivero‐Rodríguez, Isabel Cruz‐Gallardo, Christian Grüttner, Marian Hernández‐Vellisca, Irene Díaz‐Moreno

**Affiliations:** ^1^ Instituto de Investigaciones Químicas (IIQ) Centro de Investigaciones Científicas Isla de la Cartuja (cicCartuja) Universidad de Sevilla Consejo Superior de Investigaciones Científicas (CSIC) Sevilla Spain; ^2^ King's College London Randall Division of Cell & Molecular Biophysics London UK

**Keywords:** apicoplast RNA binding protein, malarial *Plasmodium* parasite, protein aggregation

## Abstract

Malaria is caused by Apicomplexa protozoans from the *Plasmodium* genus entering the bloodstream of humans and animals through the bite of the female mosquitoes. The annotation of the *Plasmodium vivax* genome revealed a putative RNA binding protein (apiRBP) that was predicted to be trafficked into the apicoplast, a plastid organelle unique to Apicomplexa protozoans. Although a 3D structural model of the apiRBP corresponds to a noncanonical RNA recognition motif with an additional C‐terminal α‐helix (α_3_), preliminary protein production trials were nevertheless unsuccessful. Theoretical solvation analysis of the apiRBP model highlighted an exposed hydrophobic region clustering α_3_. Hence, we used a C‐terminal GFP‐fused chimera to stabilize the highly insoluble apiRBP and determined its ability to bind U‐rich stretches of RNA. The affinity of apiRBP toward such RNAs is highly dependent on ionic strength, suggesting that the apiRBP–RNA complex is driven by electrostatic interactions. Altogether, apiRBP represents an attractive tool for apicoplast transcriptional studies and for antimalarial drug design.

**Abbreviations**
apiRBP: apicoplast RNA binding protein
ARE: AU‐rich element
CD: circular dichroism
DDM: n‐dodecyl‐β‐D‐maltoside
DTT: dithiothreitol
ER: endoplasmic reticulum
IPTG: isopropyl‐β‐D‐thiogalactopyranoside
ITC: isothermal titration calorimetry
LB: Luria–Bertani
MD: molecular dynamics
NLS: nuclear localization signal
PATS: prediction of apicoplast‐targeted sequences
PlasmoAP: *Plasmodium* apicoplast prediction
PMSF: phenylmethylsulfonyl fluoride
RBD: RNA binding domain
RBP: RNA binding protein
RNP1: ribonucleoprotein consensus sequence 1
RNP2: ribonucleoprotein consensus sequence 2
RRM: RNA recognition motif
SP: signal peptide
TP: transit peptide
U‐rich: uridine‐rich

Malaria is one of the most devastating parasitic diseases in the world, causing death to 1–2 million people per year, mostly children. This disease is caused by Apicomplexa protozoans from the *Plasmodium* genus passing onto humans and animals through the bite of the female mosquitoes from the *Anopheles* genus 1. The discovery of an essential plastid organelle, the so‐called apicoplast, has rekindled current search for new drugs to fight malaria 2. Widely found in Apicomplexa except for *Cryptosporidium* species 3, the apicoplast is a vestigial nonphotosynthetic plastid surrounded by four membranes due to its secondary endosymbiosis origin. Indeed, an ancient eukaryotic cell engulfed a cyanobacterium to become a photosynthetic eukaryotic alga. Then, a *Plasmodium* predecessor ingested the eukaryotic alga to establish a new symbiosis and preserved it as a plastid 4. Hence, the prokaryote‐derived metabolic pathways within the apicoplast are substantially different to those from the human host, bringing new opportunities to design drugs against malaria 5.

The apicoplast genome of *Plasmodium* spp. consists of a highly conserved ~ 35‐kb circular, double‐stranded DNA lacking the genes encoding proteins involved in photosynthesis 6. Unlike mRNAs produced in the parasite nucleus, those synthesized in the apicoplast are polycistronic, and they mainly provide the essential machinery for transcription and translation needed for organelle housekeeping functions 7. An important exception is the presence of the SufB protein (ycf24), which is a constituent of the [Fe–S] biogenesis pathway 8. To our knowledge, a small number of proteins involved in transcription, control of mRNA stability, and translation within the organelle have been studied 7, 9, 10.

Many genes initially encoded in the apicoplast have been transferred to the parasite nucleus to avoid deleterious mutations of nonrecombinant genomes. Consequently, apicoplast biogenesis and function critically relies on targeting nuclear encoded proteins back to the organelle, by distinctive apicoplast targeting motifs. Apicoplast proteins present a bipartite leader sequence at its N terminus consisting on a hydrophobic signal peptide (SP) and a chloroplast‐like transit peptide (TP). While the SP allows the entry into the secretory pathway, the TP is a relatively simple and flexible trafficking signal for post‐translational targeting and translocation to the apicoplast 2, 11 (Fig. 1).

**Figure 1:**
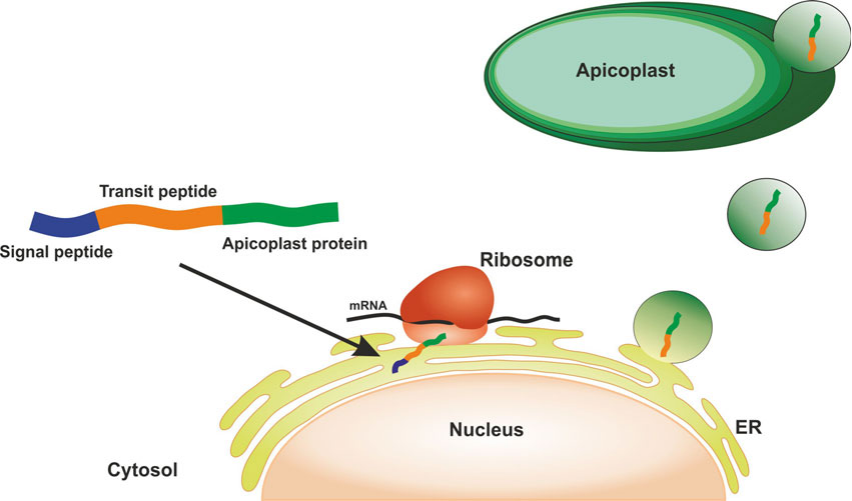
Protein targeting to the *Plasmodium* spp. apicoplast. Most apicoplast proteins traffic to the organelle due to N‐terminal bipartite leaders (SP and TP). The nascent apicoplast proteins are targeted to the endoplasmic reticulum (ER) membrane, where its SP is removed during cotranslation by a signal peptidase. The exact mechanisms that lead the transport from the ER lumen to the apicoplast are not fully understood but may involve ER–apicoplast communication by vesicular transport and TP recognition 2.

Up to 466 apicoplast predicted proteins were identified in the *P. falciparum* genome using the PlasmoAP and PATS algorithms that extract amino acid features from TP that target proteins to the organelle 12, 13. A similar analysis in the *P. vivax* genome revealed the presence of 316 proteins predicted to be targeted to the apicoplast 14, 15. One of them (PVX_084415 or apiRBP in this manuscript) is a putative RNA binding protein (RBP) displaying a predicted N‐terminal signal peptide and a single RNA recognition motif (RRM). In *P. falciparum*, 189 genes have been annotated as putative RBPs: 179 of them possess an ortholog in *P. vivax*, including the one coding apiRBP 16. Unfortunately, most of them lack definitive functional annotations and only a few have been structurally characterized (e.g., PDB: 2N7C and 2MYF).

To unveil fundamental aspects of RNA metabolism in *Plasmodium* that may hint to new targets for antimalarial therapy, we took the challenge of characterizing apiRBP, which is extremely prone to aggregation. In this work, we resorted to theoretical solvation analysis of a 3D model of the protein to design a soluble GFP‐fused chimera. Finally, we proved by calorimetry assays that apiRBP is indeed able to recognize target RNA stretches, presumably driven by electrostatic interactions.

## Materials and methods

### apiRBP plasmid constructs

**Figure 2:**
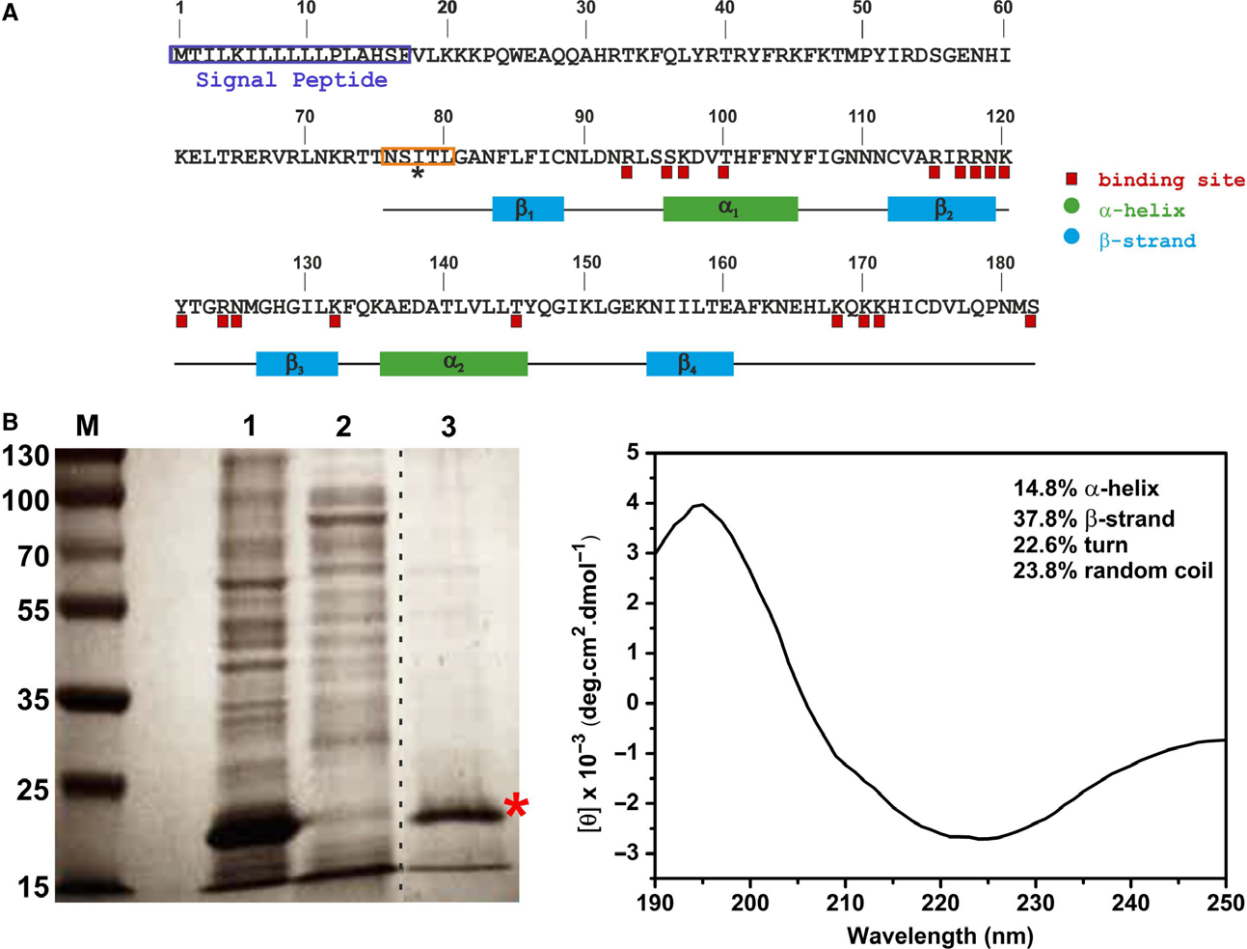
apiRBP amino acid sequence, solubilization from inclusion bodies and secondary structure characterization. (A) apiRBP full‐length sequence along with the constructs used in this study: His‐apiRBP* (residues 76–182) and His‐apiRBP (residues 81–182). The five N‐terminal amino acids that are included in the first construct, but not in the second, are marked by an orange square and an asterisk. The apiRBP SP is colored in purple. Predicted secondary structure elements by JPred V.4 30 (α‐strand and β‐sheet) and predicted RNA binding sites by BindN software 31 are also indicated. (B) (*Left*) SDS/PAGE Coomassie gel (12%) of His‐apiRBP* (residues 76–182) expression in *E. coli*. M stands for molecular mass marker in kDa. Lanes 1 and 2 are the pellet and the supernatant from cell sonication, while lane 3 stands for the supernatant of inclusion bodies refolding. The protein was found in fractions from lanes 1 and 3 (marked with a red asterisk) with a molecular weight of 19 kDa. All lanes come from the same gel, but it has been spliced and put together (dashed line between lanes 2 and 3). (*Right*) Far‐UV (190–250 nm) CD spectrum of the refolded protein indicating the contents of secondary structure elements.

Three different apiRBP plasmid constructs were used in this study. The first one, His‐apiRBP* (residues 76–182), was obtained by amplification of the PVX_084415 gene from *P. vivax* Sal1 cDNA and was cloned into the NcoI–NotI cloning sites of the pETM‐11 vector (Invitrogen) for protein expression in *Escherichia coli*. The second one, His‐apiRBP (residues 81–182), is a derivative construct from His‐apiRBP* but lacking the first five amino acids (NSITL) (Fig. 2A). It was cloned into the pIVEX2.4d cell‐free expression vector by the In‐Fusion cloning kit (Clontech) following the manufacturer's instructions. The third construct, apiRBP‐GFP‐His, was cloned in a modified pET21a(+) vector. Such vector was kindly provided by Prof. Frank Bernhard (Frankfurt, Germany) and contains a C‐terminal GFP‐His‐tag (superfolderGFP) as a quantitative reporter of gene expression. In this variant, the apiRBP gene also comprehends residues 81–182. All primer sequences are available upon request.

### Protein expression

His‐apiRBP* (residues 76–182) recombinant protein was expressed in Luria–Bertani (LB) medium in *E. coli* BL21 (DE3) cells. Cultures were grown at 37 °C with continuous agitation of 180 r.p.m. Protein expression was induced by the addition of isopropyl‐β‐D‐thiogalactopyranoside (IPTG) to a final concentration of 1 mm once an OD_600_ of 0.8 was reached. Cells were collected by centrifugation after 5 h of continuous agitation at 30 °C. To isolate the inclusion bodies, cells obtained from 1 L of culture were resuspended in 20 mL of 100 mm Tris/HCl buffer (pH 8) with 100 mm NaCl, 1 mm dithiothreitol (DTT), and 1 mm phenylmethylsulfonyl fluoride (PMSF). Lysozyme was added to the cell suspension (0.35 mg·mL^−1^), which was then sonicated. After that, the suspension was treated with DNase I (20 mg·mL^−1^) for 1 h at 37 °C and centrifuged at 30 000 ***g*** for 15 min at 4 °C. The pellet, containing the inclusion bodies, was washed twice with ice‐cold PBS buffer enriched in 1% of Triton X‐100. The inclusion bodies were then resuspended in 2 mL of 100 mm Tris/HCl ice‐cold buffer (pH 8) containing 100 mm NaCl, 1 mm DTT, and 6 m guanidinium chloride. The suspension was incubated at 20 °C for 2 h and centrifuged at 100 000 ***g*** for 20 min at 4 °C. The inclusion bodies were soluble in the supernatant fraction, which mainly contained the recombinant His‐apiRBP*, so protein refolding was performed without further purification. Two milliliters of inclusion bodies was added to 200 mL of ice‐cold refolding buffer containing 100 mm Tris/HCl (pH 8), 5 mm EDTA, and 0.5 m L‐arginine with low agitation at 15 °C 17. After 24‐h incubation at 15 °C with continuous agitation, the protein solution was centrifuged and most of the expressed His‐apiRBP* was soluble in the supernatant which was dialyzed against 100 mm Tris/HCl (pH 8) buffer.

His‐apiRBP (residues 81–182) was expressed by cell‐free protein synthesis. For this purpose, 50 μL of reaction in batch mode was produced. Briefly, 10 μg·mL^−1^ of His‐apiRBP in pIVEX2.4d was added to the reaction mixture. The reaction mixture consisted of 1 mm amino acid mix, 0.8 mm ribonucleotides (guanosine‐, uracil‐, and cytidine‐triphosphate), 1.2 mm adenosine triphosphate, 55 mm HEPES, pH 7.5, 68 μm folinic acid, 0.64 mm cyclic adenosine monophosphate, 3.4 mm dithiothreitol, 27.5 mm ammonium acetate, 2 mm spermidine, 80 mm creatine phosphate, 208 mm potassium glutamate, 16 mm magnesium acetate, 250 μg·mL^−1^ creatine kinase, 27 μg·mL^−1^ T7 RNA polymerase, 0.175 μg·mL^−1^ tRNA, and 67 μL·mL^−1^ S30 *E. coli* bacterial extract. Incubation was carried out at 23 °C with agitation for 16 h.

Finally, the apiRBP‐GFP‐His chimera was produced in LB medium in *E. coli* BL21 (DE3) cells. Cultures were grown at 37 °C with continuous agitation of 180 r.p.m. Protein expression was induced by the addition of IPTG to a final concentration of 0.5 mm after reaching OD_600_ of 0.3. Cells were collected by centrifugation after 16 h of continuous agitation at 20 °C. Posterior to cell disruption by sonication in the presence of 1 mm PMSF and cOmplete Protease Inhibitors (one tablet per 50 mL extraction solution; Sigma), extracts were centrifuged at 17 000 ***g*** and the His‐tagged protein was then purified by nickel affinity chromatography (Ni Sepharose 6 Fast Flow; GE Healthcare) applying an imidazole gradient (10–300 mm) in 20 mm Tris and 100 mm NaCl, pH 7.4. The purified fractions containing apiRBP‐GFP‐His were then submitted to FPLC (AKTA Prime) to remove protein contaminants. Protein concentrations were determined spectrophotometrically by the Bradford assay, and the molecular weight of the constructs was verified by MALDI‐TOF spectroscopy.

### Circular dichroism spectroscopy

Circular dichroism (CD) spectrum was recorded on a Jasco J‐815 spectropolarimeter equipped with a Peltier temperature control system. The secondary structure analysis of His‐apiRBP* (residues 76–182) was performed by recording far‐UV CD spectra (190–250 nm) with 3 μm samples in H_2_O at 25 °C. The spectrum was an average of 20 scans. The α‐helix and β‐sheet content was obtained with CDPRO software 18, which includes the algorithms CONTIN, SELCON and CDSSTR and the CLSTR option to compare the protein folding with a set of similar folded proteins.

### Isothermal titration calorimetry

Isothermal titration calorimetry (ITC) experiments were performed using a microcalorimeter (TA Instruments) at 25 °C titrating apiRBP‐GFP‐His or GFP‐His (10 μm) over 10‐mer U‐rich RNA oligonucleotides (1 μm; Sigma). Both the proteins and oligonucleotide samples were in 20 mm Tris (pH 7.4) buffer with/without 50 mm NaCl (indicated in each experiment). Measurements were repeated at least twice. The integrated data of heat per injection normalized per mol of injectant versus molar ratio were analyzed with AFFINIMETER software v2.1608 and were fitted to a 1 : 1 interaction model.

### Structural model

The 3D model for apiRBP (81–182) was built with Ginzu and Rosetta 19, 20 as implemented in the Robetta server (http://robetta.bakerlab.org). The Ginzu prediction assigned the sequence to a cluster of RRM‐containing proteins, whose reference parent is the histone‐lysine N‐methyltransferase SETD1A (Chao *et al*., unpublished, PDB: 3S8S).

### Molecular dynamics computations

Molecular dynamics (MD) computations were carried out in a periodic orthorhombic box using AMBER12 with the AMBER 14SB force field 21 and PME electrostatics with a Ewald summation cutoff of 9 Å. For this purpose, the system was neutralized with seven Cl^−^ ions and solvated with 6058 OPC 22 water molecules. The protein model side chains were relaxed by energy minimization. Then, solvent and counter‐ions were subjected to 5000 energy minimization steps followed by 500‐ps NPT‐MD computations using isotropic molecule position scaling and a pressure relaxation time of 2 ps at 298 K. Temperature was controlled by a Langevin thermostat 23 with a collision frequency of 5 ps^−1^. The density of the system reached a plateau during the first 50 ps. Then, the whole system was energy‐minimized and submitted to NVT‐MD computations at 298 K. The SHAKE algorithm 24 was used to constrain bonds involving hydrogen atoms. Trajectory analyses, including grid inhomogeneous solvation theory (GIST) 25 analyses, were carried out with CPPTRAJ 26. Grid spacing was 0.5 Å. UCSF Chimera 27 was the software used for molecular graphics and modeling interface.

## Results

### apiRBP annotation and secondary structure

A search of the apiRBP gene (transcript ID PVX_084415) in PlasmoDB (the official database of *Plasmodium* sequencing projects 28) predicted an RNA binding protein (RBP) of 182 amino acids. In its N‐terminal end, a 17‐residue SP was also annotated by combining the predictions of SignalP 3.0 with orthology information (Fig. 2A). However, the plastid TP of apiRBP has not been bounded yet, probably due to the fact that they are normally variable in length, have no primary consensus sequence, and are only distinguished by positively charged residues and an abundance of hydroxylated residues 29. The InterPro Domain search revealed that apiRBP contains a single RNA recognition motif (RRM) of about 90 amino acids whose secondary structure analysis by JPred V.4 30 corresponded to the canonical β_1_α_1_β_2_β_3_α_2_β_4_ topology, with four‐stranded β‐sheets packed against two α‐helices. Different lengths of the RRM are proposed, depending on the superfamily taken as a reference; for instance, SSF54928 superfamily aligns with residues 75–169, while the PS50102 Prosite domain aligns with residues 83–163. Hence, different construct lengths were tested in this report (see below). BindN software 31 also identified several potential RNA‐binding residues within the RRM taking into account the p*K*_a_ value, hydrophobicity index, and molecular mass of each amino acid. Of note, among those binding residues, is the prevalence of positively charged residues (Arg and Lys stretches), especially the ones localized in the β_2_‐strand and the C‐terminal tail (Fig. 2A).

His‐apiRBP* (residues 76–182, more alike to the SSF54928 superfamily) was produced in *E. coli* cells and isolated from inclusion bodies due to its low solubility in the supernatant fraction (Fig. 2B, *left*). Correct folding and secondary structure content were assessed by CD (Fig. 2B, *right*). The secondary structure content was 14.8% α‐helix, 37.8% β‐strand, 22.6% turn, and 23.8% random coil, in agreement with the expected values for an RRM domain, although the α‐helix content was slightly higher than expected 32, 33. Unfortunately, protein stability was too low to perform further functional assays. To test whether such instability was caused by the presence of the end of the TP in the N terminus of the apiRBP construct, we designed a shorter construction excluding amino acids 76–80, His‐apiRBP (residues 81–182, more alike to the PS50102 Prosite domain) (Fig. 2A); and cloned it in a vector compatible with cell‐free protein expression. An increase in protein solubility was achieved, still insufficient (with or without detergents) to allow the structural and functional characterization of the protein under any of the conditions tested (Fig. S1).

### apiRBP structural modeling

**Figure 3:**
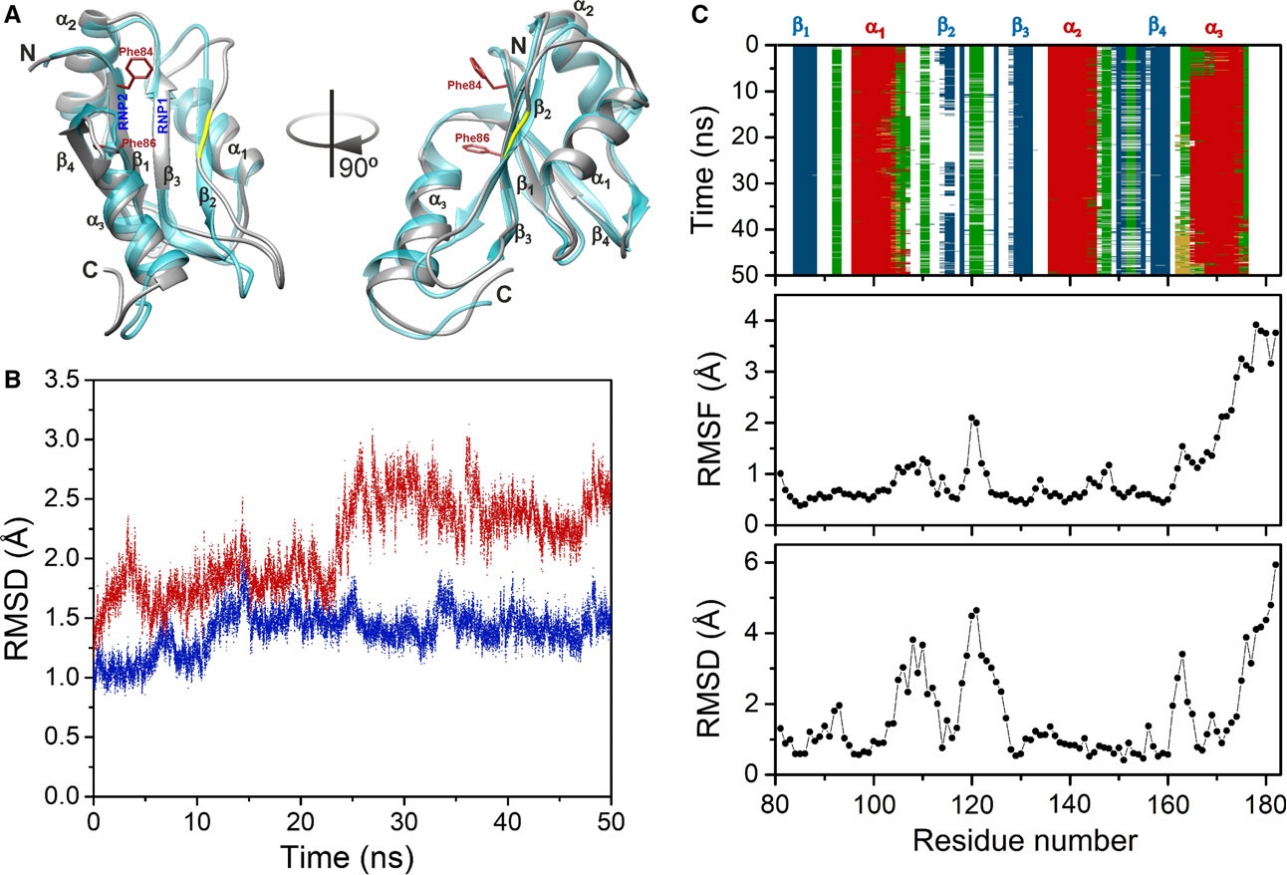
apiRBP structural modeling. (A) Overlay of the apiRBP structural model obtained with Robetta (cyan) (http://robetta.bakerlab.org) and the average one (gray) from MD computations in ribbon representation (residues 81–182). The side chains of aromatic residues of the β_1_‐sheet (Phe84 and Phe86) are displayed in red sticks. The residues in β_2_ that are absent in the sequences of homologous RRMs are highlighted in yellow. The location where the RNP1 and RNP2 consensus sequences should appear is also marked. Each view is rotated 90° around the vertical axis of the molecule. (B) Time course of RMSD values for the MD trajectory. Data corresponding to the full model (residues 81 to 182) are depicted in red and those computed for the canonical RRM domain (residues 81 to 161) in blue. (C) (*Upper panel*) Secondary structure time course along the MD trajectory. β‐Strands are in blue, bends in gray, turns in green, 3_10_‐helices in ocher, and α‐helices in red. (*Center panel*) Per‐residue atomic fluctuations along the last 10 ns of the trajectory. (*Bottom*) Per‐residue backbone RMSD computed between the model and the average structure from MD.

A 3D structural model for apiRBP was obtained by the comparative methods implemented in the Robetta server (http://robetta.bakerlab.org) (Fig. 3A). The structural model displayed the expected β_1_α_1_β_2_β_3_α_2_β_4_ topology of canonical RRMs followed by an extra α‐helix (herein named as α_3_) using the structure of the histone‐lysine N‐methyltransferase SETD1A (PDB: 3S8S) and other RRM‐containing proteins as templates. Moreover, a structural homology search using DALI database 34 returned a Z‐score of 14.4, with an RMSD value of 1.8 Å, between apiRBP and SETD1A, including the novel C‐terminal α_3_‐helix found in apiRBP. Comparison of apiRBP with another RBPs, such as ELAV‐like protein 1 (PDB: 4FXV), returned a Z‐score of 12.3, although DALI consistently excluded the α_3_‐helix of apiRBP from such structural alignments. The 10‐residue α_3_‐helix is oriented parallel with respect to the β‐sheet, mainly through contacts between Leu167 at α_3_ and the β‐sheet face (Phe86 in β_1_ and Ile130 in β_3_), along with the H‐bond involving Asn164 (α_3_)‐O_δ1_ and Arg117 (β_2_)‐guanidinium groups. Additionally, the sequence of apiRBP shows a two‐residue insertion at β_2_ (Ala114 and Arg115) with respect to homologous RRMs such as those from ELAV‐like protein 1 (31.2% identity; PDB: 4FXV), the RNA recognition motif 1 from HuR (31.2% identity; PDB: 3HI9), or RNA binding domain 1 from HuC (31.9% identity; PDB: 1D8Z). However, the secondary structure of β_2_ seems to be unaffected in the model. Interestingly, the ribonucleoprotein consensus sequences (RNP2: [ILV]‐[FY]‐[ILV]‐X‐N‐L and RNP1: [RK]‐G‐[FY]‐[GA]‐[FY]‐[ILV]‐X‐[FY]) of RRM domains at the β_1_‐ and β_3_‐strands 33 are not conserved in apiRBP showing only two aromatic residues (Phe84 and Phe86) at the RNP2 of the β1‐strand (Fig. 3A). Further, the loop joining β_4_ and α_3_ covers the aromatic ring of Phe86, probably preventing it to form π‐stacking interactions with RNA.

To check the behavior of β_2_ and α_3_, and to understand the experimentally observed instability of the protein, a 50‐ns molecular dynamics computation was reckoned (Fig. 3). The RMSD evolution depended on whether residues 81–161 or 81–182 were used for the structural alignments (Fig. 3B). This indicates that the C‐terminal end of apiRBP—including α_3_—is more mobile than the canonical RRM core. The secondary structure timeline (Fig. 3C) shows that the structure of α_3_ is stable along the trajectory. Thus, the RMSD differences in Figure 3B can be attributed to a slight rigid‐body motion of α_3_ and the high mobility of the last C‐terminal residues (stretch 176–182). Interestingly, the three‐amino acid sequence (from 162 to 164) neighboring the N terminus of α_3_‐helix is folded in a short 3_10_‐helix along MD (highlighted in ocher in Fig. 3C). Consistently, residues 162–182 show the highest fluctuations and RMSD values when comparing the average structure to the initial, energy‐minimized model (Fig. 3C). In addition, β_2_ resulted in being unstable probably because of the above‐mentioned two‐residue insertion (highlighted in yellow in Fig. 3A) with respect to other RRMs. Notably, the sequence stretches flanking the insertion showed high fluctuations and RMSD values with respect to the initial, energy‐minimized model (Fig. 3A and 3C).

### Solvation analysis of apiRBP

**Figure 4:**
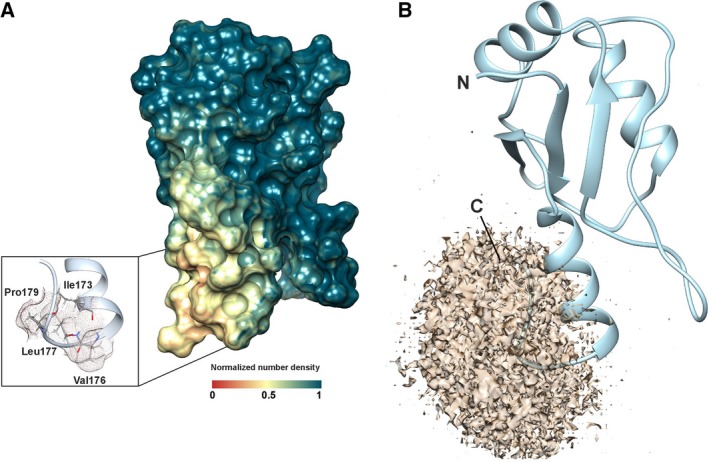
apiRBP solvation analysis. Number density of water oxygen centers across simulations, normalized with respect to bulk values and calculated by GIST implemented in CPPTRAJ. (A) Map of normalized number density values at the surface of the molecular model. The inset shows the hydrophobic cluster at C‐end α_3_. (B) Limit surface for the region showing normalized number densities lower than 0.35 voxel^−1^.

To understand the high propensity of apiRBP to aggregate in solution, MD computations were performed with explicit OPC water molecules, which are the best to represent the bulk properties of water 22. Then, the trajectories were analyzed using the grid inhomogeneous solvation theory to assess the number density of water molecules around the domain (Fig. 4A and 4B). Notably, the density of water molecules decreased near the surface patch of the RRM, and the translational entropy of water molecules in this region increased with respect to the bulk. This revealed a hydrophobic cluster near the C terminus involving side chains from Ile173 at α_3_ and Val176, Leu177, and Pro179.

### apiRBP binding to U‐rich RNA stretches

**Figure 5:**
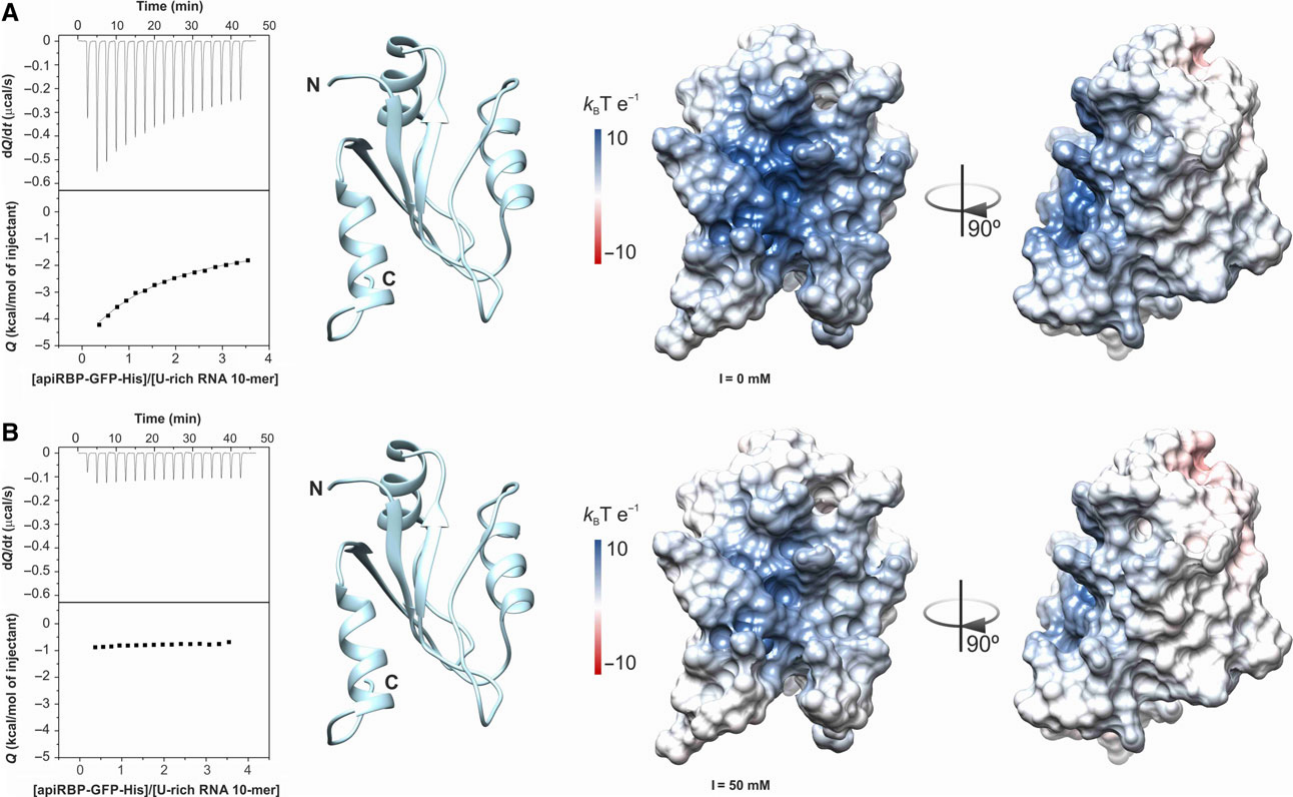
apiRBP binding to target U‐rich RNA oligonucleotides by isothermal titration calorimetry and electrostatic potential surface. (A) (*Left*) 10‐mer U‐rich RNA (1 μm, in the calorimetric cell) was titrated with apiRBP‐GFP‐His (10 μm, in the syringe) in 20 mm Tris (pH 7.4) buffer. (B) (*Left*) 1 μm 10‐mer U‐rich RNA was titrated with 10 μm apiRBP‐GFP‐His in 20 mm Tris (pH 7.4) buffer containing 50 mm NaCl. Each graph shows a thermogram of the heat released versus time in the upper panel and the integrated peak intensities plotted against the molar ratio of the complex in the lower panel. All experiments were conducted at 25 °C. (A) and (B) (*Center* and *right*) Ribbon representation and electrostatic potential surface with a color ramp for positive (blue) and negative (red) potentials at low (A) and higher (B) ionic strength values. Each view is rotated 90° around the vertical axis of the molecule. I = ionic strength.

Taking into account the results from the solvation analysis, a new version of the apiRBP construct (residues 81–182) was designed in‐frame with a C‐terminal GFP‐His‐tag (namely apiRBP‐GFP‐His). Presumably, the high stability and solubility of the GFP‐tag would serve as a stabilizing factor for the insoluble apiRBP, as previously seen for other protein targets 35. As anticipated, when recombinant apiRBP‐GFP‐His expression was performed in *E. coli*, the chimera was stable enough to perform further binding analysis (Fig. S2). As no RNA binding activity has been reported for the structurally apiRBP‐related SETD1A RRM domain 36, 37, nor for its yeast homologous SET1 RRM1 38, in this work we tested the ability of apiRBP to bind uridine‐rich (U‐rich) 10‐mer RNA. This is a consensus AU‐rich element (ARE) at the 3′‐UTR of mRNAs recognized by ELAV family of proteins that share ~ 31% of sequence identity with apiRBP. Isothermal titration calorimetry (ITC) performed at low ionic strength showed that apiRBP‐GFP‐His recognized 10‐mer U‐rich RNA stretches (Fig. 5A**,** *left*) with an affinity within the μm range when fitting the stoichiometry to *n* = 1 (Table 1). This indicates that one molecule of apiRBP‐GFP‐His binds to one molecule of RNA (1 : 1). When 50 mm of NaCl was added to the ITC buffer, the recognition of 10‐mer U‐rich RNA was negligible (Fig. 5B, *left*). Beyond the high isoelectric point (p*I*) of apiRBP (p*I* = 9.72), its surface electrostatic potential at low ionic strength (Fig. 5A**,** *center* and *right*) showed a high positively charged face (surrounding RRM β_2_ and α_3_) and a slightly negative patch in the opposite side of the protein. This evidences a dipole, which is partly attenuated at higher ionic strength (Fig. 5B**,** *center* and *right*), although no substantial differences in His‐apiRBP solubility have been observed upon salt addition (data not shown). The strong dependence on ionic strength of the binding indicated that the apiRBP–RNA complex is mainly driven by electrostatic interactions rather than π‐stacking. This is consistent with the fact that aromatic residues placed at RNP2 are occluded by the extra α_3_‐helix. However, we cannot exclude that binding to other RNA/DNA stretches would lead to higher protein‐target affinities and/or sequence specificity. Finally, a negative control carried out with free GFP‐His showed no interaction by ITC with 10‐mer U‐rich RNA, proving that apiRBP is leading RNA recognition. Additionally, the apiRBP‐GFP‐His dilution experiment in 20 mm Tris (pH 7.4) produced no substantial heats (Fig. S3).

**Table 1:** Equilibrium and thermodynamic parameters for the interaction of apiRBP‐GFP‐His affinity with 10‐mer U‐rich RNA. Relative errors corresponding to the standard deviation of the fitting: *K*_D_, 20%; ΔH and −TΔS, 5%; ΔG, 2%

*K*_D_ (μm)	3.36
*n*	1
ΔH (kcal·mol^−1^)	−2.04
ΔG (kcal·mol^−1^)	−7.5
−TΔS (kcal·mol^−1^)	−5.42

## Discussion

The cyanobacterial origin of the apicoplast makes this organelle an excellent target for pharmaceutical research 39. Here, we have characterized a putative *P. vivax* RBP that is predicted to be targeted to the apicoplast 14, 15. The elevated protein conservation of its orthologous in the genus *Plasmodium* (72% of amino acid similarity) indicates that this protein may play a key role for the malaria parasite life cycle. Most of RBPs are composed of small RNA binding domains (RBDs) that are needed for their recruitment to specific RNA targets. Within RBDs, there are four prominent families: RNA recognition motifs (RRMs), zinc fingers, KH domains, and double‐stranded RNA binding motifs 33.

The identification of secondary structure elements in apiRBP corresponded with a noncanonical RRM domain (four‐stranded β‐sheet packed against two α‐helices), which is extended by a C‐terminal α_3_‐helix. This is consistent with the increased α‐helix content detected by CD (14.8%) in comparison with other RRMs 32. Other noncanonical RRMs that include additional secondary elements have been extensively reported in the literature. Such is the case of the extra N‐terminal α‐helix (α_0_) in the third RRM of TIA‐1 (RRM3) 40, 41, the β‐sheet extended by an additional fifth antiparallel β‐strand in PTB RRM2 and RRM3 modules 42, or the RRM with an extra C‐terminal helix (xRRM) in La and LARP7 proteins 43, 44. In addition, its RNP1 module is not fully conserved, whereas RNP2 is partly occluded by α_3_‐helix.

Eukaryotic RRMs are often found as multiple copies within a protein, and together with other protein domains, they confer different affinity and specificity for the RNA sequences 45, 46, 47, 48, 49, 50, 51, 52, 53. RRMs are also found in prokaryotes, where they tend to occur as single domains in small proteins, typically around 100 amino acids in length (Pfam ID PF0076 and InterPro ID IPR000504 54, 55). Both the short length and the presence of a single RRM domain in apiRBP point to an endosymbiotic origin of the protein.

In most of the solved RRM–RNA complexes, two of the three aromatic residues in RNP1 make stacking interactions with the nucleobases, while the third can interact hydrophobically with the sugar rings. In addition, an Arg or a Lys residue at RNP1 forms a salt bridge with the phosphodiester backbone 33. Particularly interesting in apiRBP is the lack of aromatics at the strand β_3_, suggesting that the binding between apiRBP and RNA might occur by electrostatic steering rather than by nucleobase stacking. This is in agreement with the mapping of the surface electrostatic potential showing a highly positively charged face surrounding β_2_ and the C‐terminal α_3_‐helix; and also with the finding that a higher salt concentration in the buffer disrupted the binding of apiRBP to target U‐rich RNAs by ITC. Finally, the prediction server BindN 31 localized potential RNA binding sites that were out of the limits of the strands β_1_ and β_3_ (particularly concentrated in β_2_)_._ Then, if RNA recognition was β_2_‐mediated, this highly dynamic secondary element could be fixed upon apiRBP–RNA complex formation. Examples of RRMs using unusual RNA binding surfaces are reported in the literature. In such cryptic, atypical RRMs, aromatic residues have been displaced from the β_1_–β_3_ motif to the β‐connecting loops in qRRMs 56 or to the so‐called RNP3 at β_2_ in xRRMs 43, 44, 57. In the particular case of apiRBP RRM, β_2_‐strand could bind to RNA through electrostatic contacts. Additionally, we cannot exclude the participation of the C‐terminal end of apiRBP in the binding to RNA as it is also highly positively charged. Both β_2_ and α_3_ may take part of a wide RNA binding platform. Altogether, these findings suggest an uncommon way of binding for an unusual RRM module and open a new door for further structural and functional characterization to better understand those mechanisms.

The fold of a protein and its stability toward denaturation are governed by the sequence of amino acids and the environment (solvent, salts, pH, temperature, crowding, etc.) 58. Expression of a soluble recombinant apiRBP in *E. coli* was unsuccessful under the conditions tested in this study. The solvation analysis of apiRBP showed an exposed hydrophobic cluster at the C terminus. We then resolved to fuse a GFP‐tag to the C‐terminal end of the protein generating a more stable chimera that was useful for functional assays. Indeed, a model of the apiRBP‐GFP chimera protein showed that GFP blocked the C‐end hydrophobic patch in the RBP, probably preventing aggregation and interfering, somehow, with RNA binding (Fig. S4). A recent thermodynamic and molecular modeling study revealed that reaction in tight nucleic acid–RRM interactions is mostly enthalpy‐driven 59. According to their analysis, all these interactions display a strong enthalpy–entropy compensation effect (see Fig. 4A in 59). Despite the fact of using a GFP‐tagged chimera, plotting our values in Figure 4A of the manuscript would match their fitting at one extreme. Similar compensation effects have been observed in other systems 60, 61. Nevertheless, for a deeper structural approach, it would be of great interest to study the RRM module alone under native conditions.

Additionally, the exact residue where the TP finishes and the RRM domain starts in apiRBP still remains unknown. Interestingly, a study of the TP in *Toxoplasma gondii* showed that positive charges are more influential in the N‐terminal portion of the TP, that arginine and lysine are equally suitable, and that the exact position of these charges is not important 11. The accumulation of positive charges along the β_2_‐strand and the extra C‐terminal α_3_‐helix of the RRM module is particularly interesting in apiRBP. Once proven that apiRBP is targeted to the apicoplast by cellular approaches, the possible role of those positive charges into the transit to the apicoplast organelle could be investigated. This would resemble the functioning of the single RRM from the trypanosome T*c*UBP1 RBP that behaves as a structural nuclear localization signal (NLS), alternating nuclear import and RNA binding 62. Truncations and strategic point mutations of apiRBP would help to prove or reject this theory.

Historically, malarial drug design has been focused on compounds that modulate protein function such us doxycycline 63, but recently, RNA has become a new target for pharmaceutical companies 64. Aminoglycoside antibiotics that target the RNA component of the small ribosomal subunits are being widely used for the treatment of bacterial infections. In addition, novel approaches to drug discovery are identifying potential target sites in mRNA molecules, in particular the binding sites of proteins that regulate mRNA translation or stability 64. The future molecular characterizations of proteins with a crucial role in expression of plastid genes, such as apiRBP, are a promising target for RNA‐based antimalarial drugs.

## Author contributions

IDM designed research; CG, ICG, FRR, MH, and SMGM performed research; FRR, SMGM, and ADQ analyzed the data; and SMGM, ADQ, and IDM wrote the manuscript.

## Supporting information


**Fig. S1**. Solubilization tests of His‐apiRBP synthesized in cell‐free extracts.
**Fig. S2.** Protein purification, stability, and identification of apiRBP‐GFP‐His.
**Fig. S3.** Control experiments of the apiRBP‐GFP‐His binding to RNA by isothermal titration calorimetry.
**Fig. S4.** apiRBP‐GFP structural model.
