# Supplementary material for: A putative RNA binding protein from Plasmodium vivax apicoplast
Source: FEBS Open Bio. 2017 Dec 31;8(2):177–88. doi: 10.1002/2211-5463.12351 (PMC5794462; doi:10.1002/2211-5463.12351)
Supplement: Supplementary file 1 — Fig. S1. Solubilization tests of His‐apiRBP synthesized in cell‐free extracts. Fig. S2. Protein purification, stability, and identification of apiRBP‐GFP‐His. Fig. S3. Control experiments of the apiRBP‐GFP‐His binding to RNA by isothermal titration calorimetry. Fig. S4. apiRBP‐GFP structural model. [file FEB4-8-177-s001.doc]

**Supplemental Material**

**A putative RNA Binding Protein from *Plasmodium vivax* Apicoplast**

Sofía M. García-Mauriño, Antonio Díaz-Quintana, Francisco Rivero-Rodríguez, Isabel Cruz-Gallardo, Christian Grüttner, Marian Hernández-Vellisca and Irene Díaz-Moreno

**
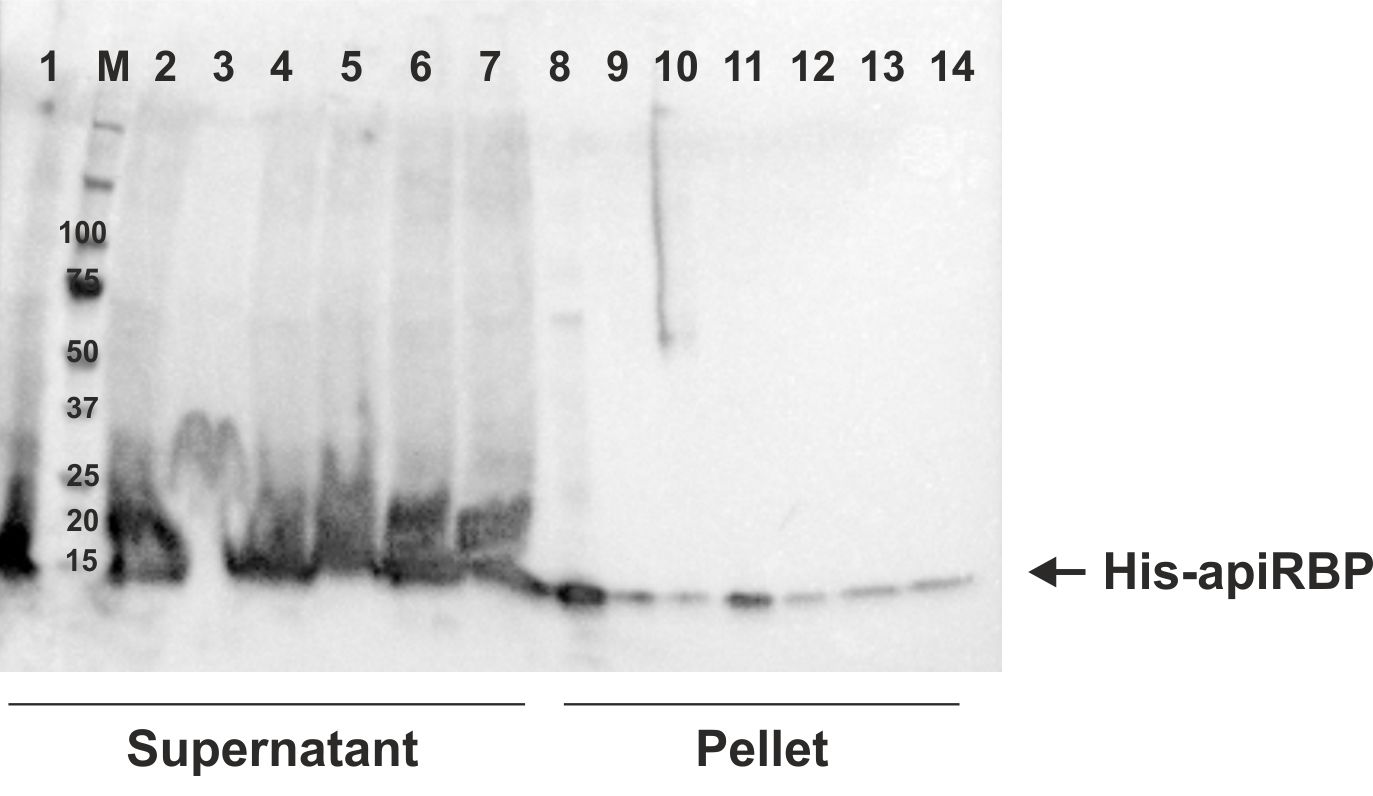
**

**Supplemental Figure 1. Solubilization tests of His-apiRBP synthesized in cell free extracts.** His-apiRBP (molecular weight of 13.3 kDa) was expressed by cell-free protein synthesis. Expression was carried out either in the absence (lane 1 and 8) or in the presence of different detergents (lanes 2-7 and 9-14). Specifically, the following detergents to a final concentration (w/v) were added: Brij 35 at 1 % (lanes 2 and 9); Brij 35 at 2.5 % (lanes 3 and 10); n-Dodecyl β-D-maltoside (DDM) at 0.08 % (lanes 4 and 11); DDM at 0.4 % (lanes 5 and 12); Brij 58 at 0.1 % (lanes 6 and 13) and Brij 58 at 0.5 % (lanes 7 and 14). The resulting material was then centrifuged and separated into supernatant and pellet fractions and run in a SDS-PAGE gel. After protein transference to a PVDF membrane, Western Blot analysis of the anti-His-tag was performed. M: molecular weight marker (kDa).


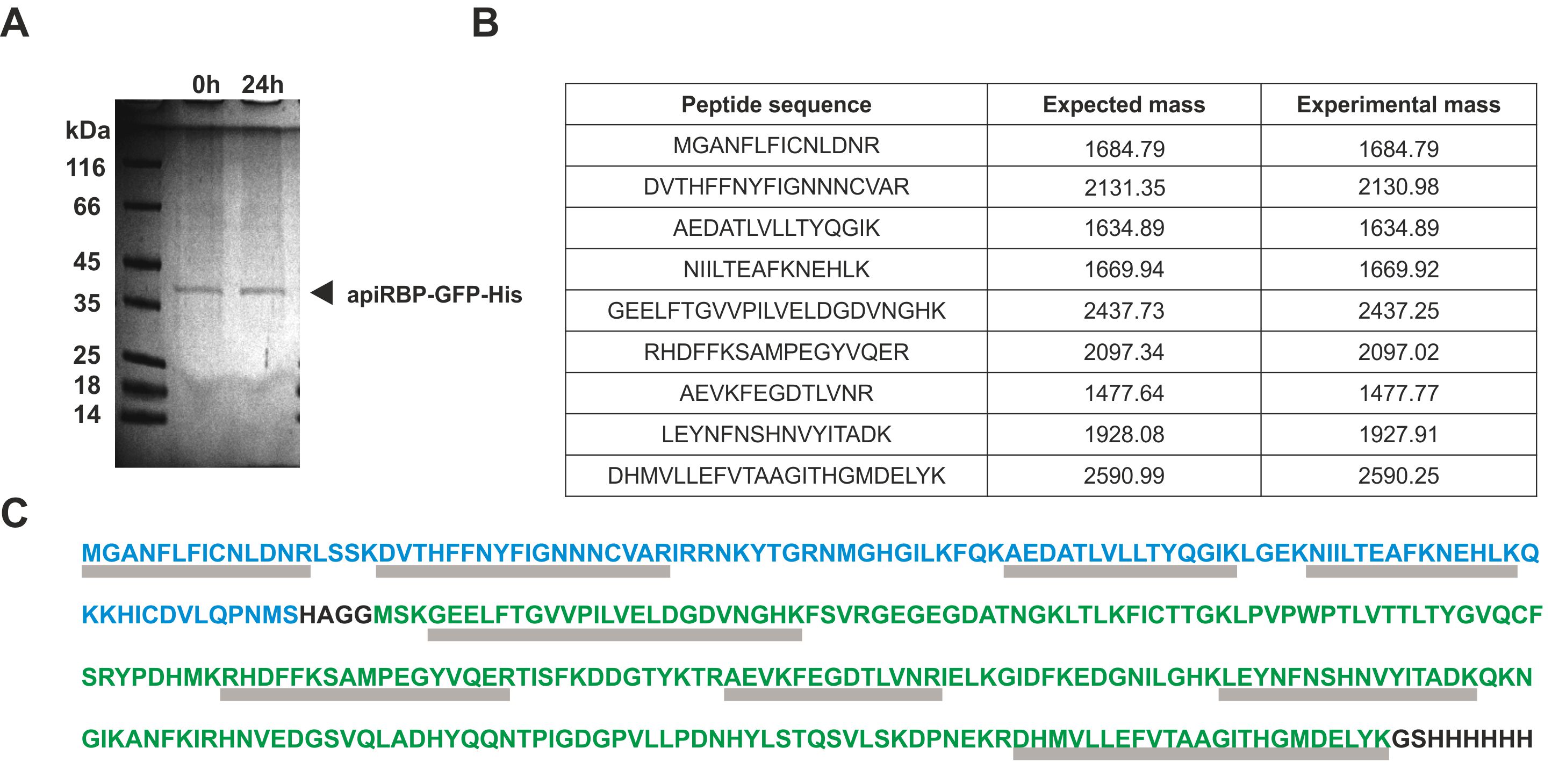


**Supplemental Figure 2. Protein purification, stability and identification of apiRBP-GFP-His. A.** SDS-PAGE gel of the full length chimera apiRBP-GFP-His (molecular weight of 39.9 kDa) after purification by FPLC (0 h) and after 24 h at room temperature (24 h). **B** and **C**. apiRBP-GFP-His peptides identified by mass spectrometry after trypsin digestion. B shows the expected and the experimental mass of the identified peptides (errors are comprehended +/- 0.5 Da) while C localizes the peptides in the chimera sequence (in grey) where apiRBP amino acids are colored in blue and GFP sequence in green.


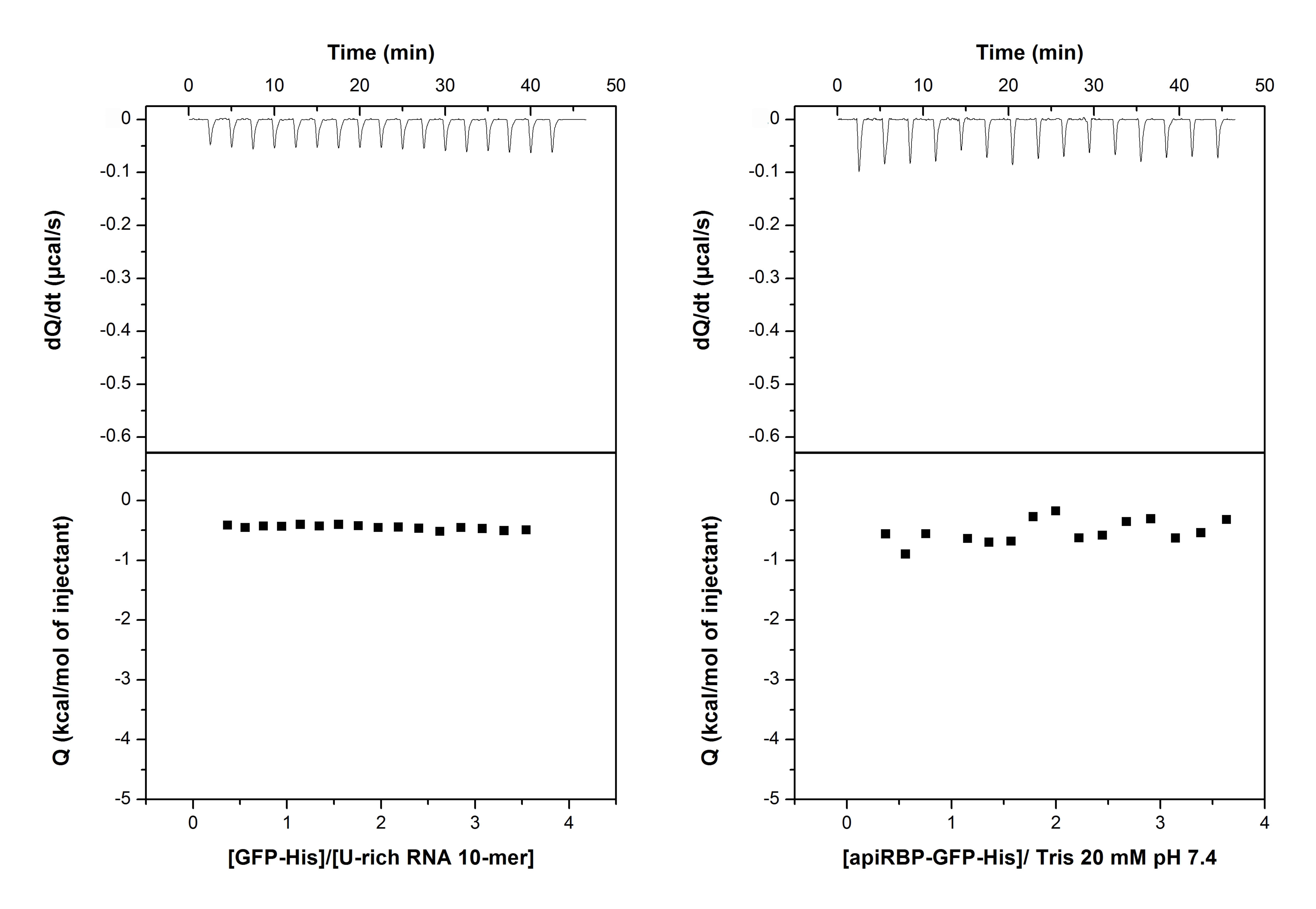


**Supplemental Figure 3. Control experiments of the apiRBP-GFP-His binding to RNA by Isothermal Titration Calorimetry.** *Left*, 10-mer U-rich RNA (1 μM, in the calorimetric cell) was titrated with GFP-His (10 μM, in the syringe). *Right*, ITC buffer was titrated with 10 μM apiRBP-GFP-His. Each plot shows the heat released versus time in the upper panel with a thermogram of the integrated peak intensities plotted against the molar ratio of the complex in the lower panel. Both experiments were conducted in 20 mM Tris (pH 7.4) at 25 ºC.


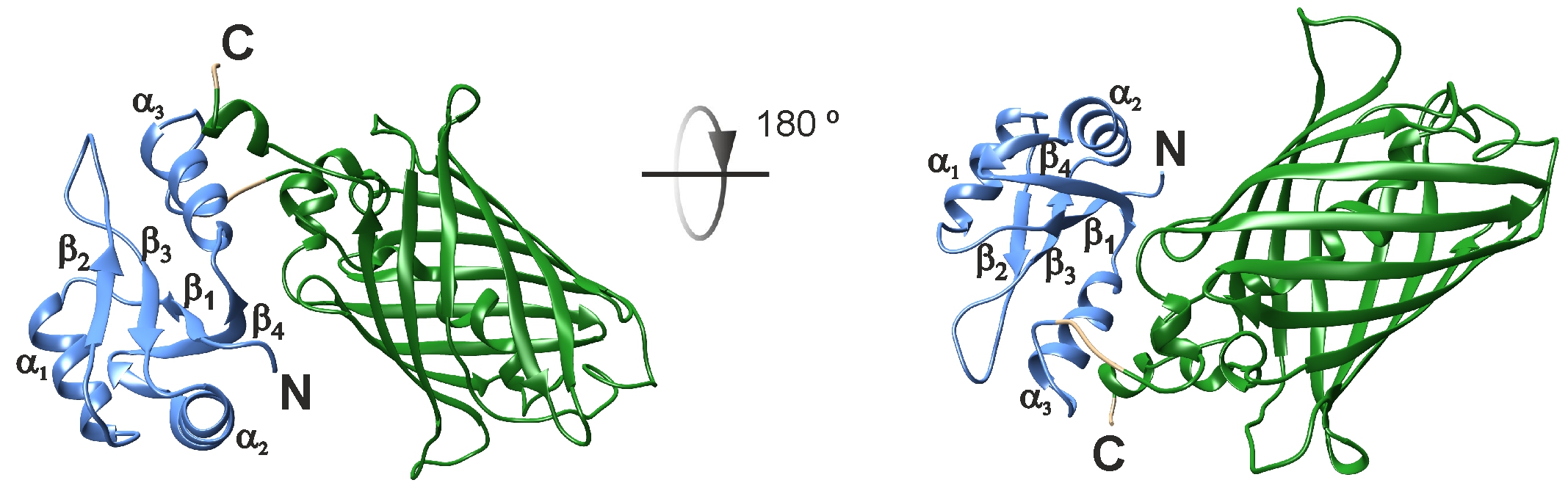


**Supplemental Figure 4. apiRBP-GFP structural model.** Model of apiRBP (in blue) with the GFP-tag (in green) obtained with Robetta ([http://robetta.bakerlab.org](http://robetta.bakerlab.org/)). Each view is rotated 180° around the horizontal axis of the molecule**.**
